# Association of Race and Area Deprivation With Breast Cancer Survival Among Black and White Women in the State of Georgia

**DOI:** 10.1001/jamanetworkopen.2022.38183

**Published:** 2022-10-28

**Authors:** Justin M. Luningham, Gaurav Seth, Geetanjali Saini, Shristi Bhattarai, Sofia Awan, Lindsay J. Collin, Monica H. Swahn, Dajun Dai, Keerthi Gogineni, Preeti Subhedar, Pooja Mishra, Ritu Aneja

**Affiliations:** 1 Department of Biostatistics and Epidemiology, School of Public Health, University of North Texas Health Science Center, Ft Worth; 2 Department of Biology, College of Arts and Sciences, Georgia State University, Atlanta; 3 School of Public Health, Georgia State University, Atlanta; 4 Department of Population Health Sciences, Huntsman Cancer Institute, University of Utah, Salt Lake City; 5 Department of Health Promotion and Physical Education, Wellstar College of Health and Human Services, Kennesaw State University, Kennesaw, Georgia; 6 Department of Geosciences, Georgia State University, Atlanta; 7 Department of Hematology–Medical Oncology, Winship Cancer Institute, Emory University School of Medicine, Atlanta, Georgia; 8 Department of Surgery, Winship Cancer Institute, Emory University School of Medicine, Atlanta, Georgia; 9 Georgia Cancer Center for Excellence, Grady Health System, Atlanta; 10 Department of Clinical and Diagnostic Sciences, School of Health Professions, University of Alabama at Birmingham

## Abstract

**Importance:**

Increasing evidence suggests that low socioeconomic status and geographic residence in disadvantaged neighborhoods contribute to disparities in breast cancer outcomes. However, little epidemiological research has sought to better understand these disparities within the context of location.

**Objective:**

To examine the association between neighborhood deprivation and racial disparities in mortality among Black and White patients with breast cancer in the state of Georgia.

**Design, Setting, and Participants:**

This population-based cohort study collected demographic and geographic data from patients diagnosed with breast cancer between January 1, 2004, and February 11, 2020, in 3 large health care systems in Georgia. A total of 19 580 patients with breast cancer were included: 12 976 from Piedmont Healthcare, 2285 from Grady Health System, and 4319 from Emory Healthcare. Data were analyzed from October 2, 2020, to August 11, 2022.

**Exposures:**

Area deprivation index (ADI) scores were assigned to each patient based on their residential census block group. The ADI was categorized into quartile groups, and associations between ADI and race and ADI × race interaction were examined.

**Main Outcomes and Measures:**

Cox proportional hazards regression models were used to compute hazard ratios (HRs) and 95% CIs associating ADI with overall mortality by race. Kaplan-Meier curves were used to visualize mortality stratified across racial and ADI groups.

**Results:**

Of the 19 580 patients included in the analysis (mean [SD] age at diagnosis, 58.8 [13.2] years), 3777 (19.3%) died during the course of the study. Area deprivation index contributed differently to breast cancer outcomes for Black and White women. In multivariable-adjusted models, living in a neighborhood with a greater ADI (more deprivation) was associated with increased mortality for White patients with breast cancer; compared with the ADI quartile of less than 25 (least deprived), increased mortality HRs were found in quartiles of 25 to 49 (1.22 [95% CI, 1.07-1.39]), 50 to 74 (1.32 [95% CI, 1.13-1.53]), and 75 or greater (1.33 [95% CI, 1.07-1.65]). However, an increase in the ADI quartile group was not associated with changes in mortality for Black patients with breast cancer (quartile 25 to 49: HR, 0.81 [95% CI, 0.61-1.07]; quartile 50 to 74: HR, 0.91 [95% CI, 0.70-1.18]; and quartile ≥75: HR, 1.05 [95% CI, 0.70-1.36]). In neighborhoods with an ADI of 75 or greater, no racial disparity was observed in mortality (HR, 1.11 [95% CI, 0.92-1.36]).

**Conclusions and Relevance:**

Black women with breast cancer had higher mortality than White women in Georgia, but this disparity was not explained by ADI: among Black patients, low ADI was not associated with lower mortality. This lack of association warrants further investigation to inform community-level approaches that may mitigate the existing disparities in breast cancer outcomes in Georgia.

## Key Points

**Question:**

Are the disparities in breast cancer survival between Black and White women across Georgia associated only with race or also with economic deprivation?

**Findings:**

In this cohort study of 19 580 patients with breast cancer, White women who lived in less deprived neighborhoods showed decreased mortality, but the same association was not observed among Black women.

**Meaning:**

These findings suggest that breast cancer mortality in Black women could be associated with additional factors related to living in economically deprived neighborhoods; considering other environmental factors can help inform community-level approaches aimed at reducing these disparities in Georgia.

## Teaser

This cohort study analyzes associations among area deprivation index, race, and mortality for Black and White women diagnosed with breast cancer in the state of Georgia in 3 large health care systems.

## Introduction

Among women in the US, breast cancer is the most commonly diagnosed cancer and is the second-leading cause of cancer death.^1^ Despite an emphasis on early detection and the availability of effective treatments, racial disparities in breast cancer mortality persist.^2,3,4^ The cause of these inequities is complex. Some factors associated with disparities in breast cancer mortality include socioeconomic status (SES), geographic residency,^2^ insurance coverage, and access to screening and care.

In the US, Black patients with breast cancer are, on average, 39% more likely to die of the disease than White patients with breast cancer.^3,4^ In the State of Georgia, this disparity is estimated at 45%.^4^ Between 2005 and 2014, the racial disparity in breast cancer mortality between White and Black women in Atlanta increased noticeably.^5^ We hypothesized that these disparities might be influenced by neighborhood deprivation, which broadly refers to the lack of economic and social resources in a neighborhood. Neighborhood factors that influence health include poverty, housing quality, unemployment,^5,6,7,8^ air pollution, tobacco marketing, water contamination, access to supermarkets, crime rates, and lack of green spaces^6,7,8^ as well as SES, which correlates with neighborhood deprivation and is known to be an important contributor to racial disparities in breast cancer outcomes.^9,10^ Cumulative and continuous exposure to neighborhood deprivation–associated stressors may partially explain why racial disparities in breast cancer outcomes persist in deprived neighborhoods: prolonged stress can induce chronic inflammation,^11,12^ which underlies most disease etiology, including cancer,^13^ and low-grade chronic inflammation biomarkers (eg, C-reactive protein and serum amyloid A protein levels) are associated with breast cancer mortality.^14,15^

Examining the association of neighborhood deprivation with breast cancer mortality may help to elucidate factors contributing to racial disparities and inform community-level approaches to mitigate these well-known disparities.^16^ In this study, we used the area deprivation index (ADI), which is computed for each census block using 17 measures from US Census data, as a measure of neighborhood deprivation.^17,18,19^ We analyzed associations among ADI, race, and mortality for Black and White women diagnosed with breast cancer in Georgia using data from 3 large health care systems.

## Methods

### Participants

In this cohort study, data from Black and White patients diagnosed with breast cancer were collected from Piedmont Healthcare, Grady Health System, and Emory Healthcare. Piedmont Healthcare includes 19 hospitals, several clinics, and hundreds of satellite locations throughout Georgia. Grady Health System supports a single large hospital in downtown Atlanta, and Emory Healthcare operates several hospitals in the Atlanta metropolitan area. The study was reviewed by the Institutional Review Board at Georgia State University, where the analyses were performed, and approved by the hospitals from which data were procured. Participant consent was waived by the institutional review board due to the nature of retrospective medical record review, in which the waiver poses minimal risk to participants, and the study could not be practically performed without the waiver because of the large number of patient records and some patients being deceased. This study followed the Strengthening the Reporting of Observational Studies in Epidemiology (STROBE) reporting guideline.

Data were collected from the patient records provided by hospital administrators. The following inclusion criteria were used to filter these records: a self-reported race of either Black or White, available home address or 9-digit zip code, and available information on overall survival time (including dates of diagnosis, dates of last contact or death, and vital status). Patient records were limited to patients who received a diagnosis up until February 11, 2020; however, we had information on some patients’ follow-ups (eg, last contact or vital status, depending on any visit to a physician in the health care system) up through July 26, 2021. After filtering, 12 976 patients from Piedmont Healthcare, 2285 from Grady Health System, and 4319 from Emory Healthcare were included.

We focused only on Black and White women for several reasons. First, we had little data on women of self-reported Hispanic ethnicity. Piedmont Healthcare did not collect information on Hispanic ethnicity; in the Grady Health System data, approximately 5% of 2285 patients self-identified as Hispanic; and in the Emory Healthcare data, approximately 2.1% of patients identified as Hispanic or Latina. To be consistent across the 3 health care systems, and because Black and White disparities are well documented and of primary interest in the Atlanta area, we included all White and Black patients (whether or not they also self-reported Hispanic ethnicity) from Grady Health System and Emory Healthcare. However, because we did not have information on ethnicity from all hospitals, we were unable to study the Hispanic population separately. In addition to these classification issues, there were demographic issues with collecting data from other races and ethnicities. In Atlanta, the Asian American and Hispanic populations tend to be younger, which may partially explain why they are underrepresented among patients with breast cancer (mean [SD] age at diagnosis was 58.8 [13.2] years in our data).

### Area Deprivation Index

The ADI is a percentile rank of socioeconomic disadvantage determined at the census block level^19^ that has been used in previous population-based studies.^20,21,22,23,24,25,26,27,28,29,30,31,32,33^ The ADI is calculated using 17 different indicators, including income level, income disparity, educational attainment, employment, home values, and quality of life. These indicators were weighted to create an underlying deprivation score.^20^ Census blocks were then ranked as a percentile (1-100) of deprivation relative to the national level; higher ADI scores indicated higher deprivation. The 5-digit zip codes obtained from patient addresses were linked to their plus-4 codes using the US Postal Service zip code database.^34^ These 9-digit zip codes were used to match patients with their census block ADI score obtained from the Neighborhood Atlas website.^35^ In the present study, ADI scores were categorized into 4 quartiles: less than 25, 25 to 49, 50 to 74, and 75 or greater.

### Clinical Variables

Survival time in months was calculated using the date of initial diagnosis and date of last contact or date of death. The most recent data extraction for the present study sample was July 26, 2021. A separate variable recorded the last known vital status of the patient. Patient records recorded all-cause deaths, not only breast cancer–caused deaths. Other clinicopathological characteristics included age at diagnosis, American Joint Committee on Cancer stage (6th, 7th, and 8th editions), tumor grade, and treatment course (chemotherapy, radiotherapy, chemoradiotherapy, or none), as well as the status of estrogen receptor (ER), progesterone receptor (PR), and *ERBB2* (formerly human epidermal growth factor receptor 2 [*HER2* or *HER2/neu*]). Patients were classified according to receptor subtype (ER positive–PR positive–*ERBB2* negative, ER positive–PR positive–*ERBB2* positive, ER negative–PR negative–*ERBB2* negative [triple negative], and ER negative–PR negative–*ERBB2* positive).

### Statistical Analysis

Data were analyzed from October 2, 2020, through August 11, 2022. Descriptive statistics were calculated first for the overall sample and then separately for Black and White patients. Multivariable Cox proportional hazards regression models were used to estimate associations of ADI and race with the time to all-cause mortality by computing hazard ratios (HRs) and their 95% CIs.

First, we examined the association between ADI and mortality in 3 models: (1) unadjusted associations, (2) ADI associations adjusted for race and age at diagnosis, and (3) ADI associations in a fully adjusted model controlling for all available clinical covariates (age at diagnosis, tumor grade, American Joint Committee on Cancer stage, treatment type, hospital system, and receptor subtype). Second, we examined racial differences in mortality estimated in 3 models: (1) unadjusted, (2) adjusted for age at diagnosis, and (3) estimated associations in the fully adjusted model. Last, we estimated a formal ADI × race interaction in the unadjusted, partially adjusted, and fully adjusted models. The models were then fitted separately for White and Black patients to compare differential associations between ADI groups and mortality across race. To avoid the “table 2 fallacy,”^36^ we provided only the HRs for the primary exposures of ADI group, race, and ADI × race interaction. This process avoids the potential for misinterpretation of numerous adjusted effects in a single model. Kaplan-Meier curves were used to visualize mortality by race and ADI group. All data cleaning, modeling, and visualization were conducted in R, versions 3.2-13 and 0.4.9 (R Foundation for Statistical Computing) using the survival package for the Cox proportional hazards regression models^37^ and the survminer package for survival curves.^38^

To examine the geographic disparities in deprivation, a continuous surface of interpolated deprivation for the study area was generated using the Empirical Bayesian Kriging method^39^ in ArcGIS. Kriging is a geostatistical method that estimates the value in a given area using existing measures nearby. The Empirical Bayesian Kriging method calculates parameters using subsets and simulations, obtaining more accurate results than other Kriging methods by accounting for the errors introduced by estimating the underlying semivariogram.^40^ Two-sided α = .05 indicated statistical significance.

## Results

### Demographics

The data set included a total of 19 580 patient records (mean [SD] age, 58.8 [13.2] years). Table 1 presents a summary of demographic information and clinical variables. Of these, 8359 patients (42.7%) across the data set identified as Black, and 11 221 (57.3%) identified as White. Demographic differences across hospital systems included 2036 of 2285 patients from Grady Health System (89.1%) who were Black, 8796 of 12 976 Piedmont Healthcare patients (67.8%) who were White, and Emory Healthcare with a near-even split of Black (2143 of 4319 [49.6%]) and White (2176 of 4319 [50.4%]) patients. Demographic differences across ADI groups included the most deprived (ADI ≥75) group with 3046 of 8359 Black women in the study (36.4%) but only 990 of 11 221 White women (8.8%), and the least deprived group (ADI <25) with 3847 of 11 221 White women (34.3%) but only 418 of 8359 Black women (5.0%). During the study period, 3777 patients (19.3%) died (all-cause mortality), but there were notable differences across racial groups: 1991 deaths were among Black women (23.8%) and 1786 were among White women (15.9%).

**Table 1.** Descriptive Statistics of Study Patient Records

Variable	Patient cohort^a^		
	Full cohort (N = 19 580)	Black (n = 8359)	White (n = 11 221)
Age at diagnosis, mean (SD), y	58.8 (13.2)	57.0 (12.9)	60.1 (13.2)
Race			
Black	8359 (42.7)	NA	NA
White	11 221 (57.3)	NA	NA
ADI^b^			
<25	4265 (22.3)	418 (5.2)	3847 (34.7)
25-49	5576 (29.1)	1711 (21.3)	3865 (34.8)
50-74	5258 (27.5)	2868 (35.7)	2390 (21.5)
≥75	4036 (21.1)	3046 (37.9)	990 (8.9)
Missing	445 (2.3)	316 (3.8)	129 (1.1)
All-cause mortality	3777 (19.3)	1991 (23.8)	1786 (15.9)
Hospital			
Emory Healthcare	4319 (22.1)	2143 (25.6)	2176 (19.4)
Grady Health System	2285 (11.7)	2036 (24.3)	249 (2.2)
Piedmont Healthcare	12 976 (66.3)	4180 (50.0)	8796 (78.4)
Hormone type			
ER positive–PR positive–*ERBB2* positive	1488 (7.6)	619 (7.4)	869 (7.7)
ER positive–PR positive–*ERBB2* negative	11 999 (61.3)	4276 (51.1)	7723 (68.8)
ER negative–PR negative–*ERBB2* positive	1133 (5.8)	569 (6.8)	564 (5.0)
ER negative–PR negative–*ERBB2* negative	3799 (19.4)	1903 (22.8)	1896 (16.9)
Missing	1161 (5.9)	992 (11.9)	169 (1.5)
Tumor grade			
1	3373 (17.2)	1042 (12.5)	2331 (20.8)
2	7358 (37.7)	2908 (34.8)	4450 (39.7)
3	8350 (42.6)	3971 (47.5)	4379 (39.0)
Missing	499 (2.5)	438 (5.2)	61 (0.5)
Cancer stage			
0	2713 (13.9)	1088 (13.0)	1625 (14.5)
1A	7198 (36.8)	2526 (30.2)	4672 (41.6)
1B	349 (1.8)	134 (1.6)	215 (1.9)
2A	3327 (17.0)	1417 (17.0)	1910 (17.0)
2B	1509 (7.7)	726 (8.7)	783 (7.0)
3A	974 (5.0)	477 (5.7)	497 (4.4)
3B	260 (1.3)	137 (1.6)	123 (1.1)
3C	302 (1.5)	151 (1.8)	151 (1.3)
4	687 (3.5)	327 (3.9)	360 (3.2)
Missing	2261 (11.5)	1376 (16.5)	885 (7.9)
Survival, mean (SD), mo	63.3 (41.6)	62.8 (43.0)	63.7 (40.6)
Cases with any missing data	3320 (17.0)	2217 (26.5)	1103 (9.8)
Missing by health care system			
Emory Healthcare	0	0	0
Piedmont Healthcare	1669 (9.0)	735 (17.6)	934 (10.6)
Grady Health System	1651 (72.0)	1482 (72.8)	169 (67.9)

Abbreviations: ADI, area deprivation index; ER, estrogen receptor; *ERBB2*, formerly *HER2* or *HER2/neu*; NA, not applicable; PR, progesterone receptor.

^a^ Unless otherwise indicated, data are expressed as No. (%) of patients. Percentages have been rounded and may not total 100.

^b^ The lowest ADI quartile (<25) indicates the lowest level of neighborhood deprivation; the highest ADI quartile (≥75) indicates the greatest level.

### Race and Clinical Variable Descriptive Statistics

Table 1 presents descriptive statistics of the clinical and pathological covariates available in the data set. In general, a higher proportion of Black patients (1903 [22.8%]) were diagnosed with the aggressive triple-negative breast cancer subtype than White patients (1896 [16.9%]). A higher proportion of Black women presented with grade 3 breast cancer tumors than White women (3971 [47.5%] vs 4379 [39.0%]), and a higher proportion of White women were diagnosed with a grade 1 tumor than Black women (2331 [20.8%] vs 1042 [12.5%]). At the time of diagnosis, the mean (SD) age of Black women was 57.0 (12.9) years, 3 years younger than the mean (SD) age of White women at diagnosis (60.1 [13.2] years). Black women were also slightly more likely to be diagnosed at later stages of the disease (eg, stage IV, 327 [3.9%] vs 360 [3.2%]). Table 1 also includes information on missing data by race, hospital, and clinical variables. The eMethods in the Supplement contains a full discussion on missing data.

### Survival Modeling

Table 2 shows results for the Cox proportional hazards regression models that examine the association of ADI group and race with mortality. As anticipated, racial disparities in survival were notable because Black women had a significant increase in mortality rate in both the unadjusted (HR, 1.49 [95% CI, 1.40-1.59]) and adjusted (HR, 1.23 [95% CI, 1.11-1.36]) models. The ADI was also significantly associated with increased mortality overall. Compared with individuals in the lowest ADI group (<25), those in the group with an ADI of 25 to 49 had an unadjusted HR of 1.33 (95% CI, 1.20-1.48) and a fully adjusted HR of  1.13 (95% CI, 1.01-1.28); those in the group with an ADI of 50 to 74 had an unadjusted HR of 1.70 (95% CI, 1.54-1.89) and a fully adjusted HR of 1.22 (95% CI, 1.08-1.39); and those in the ADI group of 75 or greater had an unadjusted HR of 2.05 (95% CI, 1.85-2.27) and a fully adjusted HR of 1.32 (95% CI, 1.14-1.53).

**Table 2.** Results of Cox Proportional Hazards Regression Models

6Model	HR (95% CI)^a^		
	Full cohort	Black patients	White patients
ADI, unadjusted			
ADI <25	1 [Reference]	1 [Reference]	1 [Reference]
ADI 25-49	1.33 (1.20-1.48)	0.89 (0.71-1.11)	1.33 (1.18-1.50)
ADI 50-74	1.70 (1.54-1.89)	0.97 (0.78-1.20)	1.83 (1.61-2.08)
ADI ≥75	2.05 (1.85-2.27)	1.15 (0.93-1.43)	2.15 (1.83-2.52)
ADI adjusted for age and race			
ADI <25	1 [Reference]	1 [Reference]	1 [Reference]
ADI 25-49	1.25 (1.12-1.38)	0.87 (0.70-1.09)	1.30 (1.15-1.46)
ADI 50-74	1.53 (1.38-1.71)	0.97 (0.78-1.19)	1.72 (1.51-1.95)
ADI ≥75	1.64 (1.46-1.84)	1.09 (0.88-1.35)	1.91 (1.63-2.23)
ADI, fully adjusted^b^			
ADI <25	1 [Reference]	1 [Reference]	1 [Reference]
ADI 25-49	1.13 (1.01-1.28)	0.81 (0.61-1.07)	1.22 (1.07-1.39)
ADI 50-74	1.22 (1.08-1.39)	0.91 (0.70-1.18)	1.32 (1.13-1.53)
ADI ≥75	1.32 (1.14-1.53)	1.05 (0.70-1.36)	1.33 (1.07-1.65)
Race, unadjusted			
White	1 [Reference]	NA	NA
Black	1.49 (1.40-1.59)	NA	NA
Race, fully adjusted^b^			
White	1 [Reference]	NA	NA
Black	1.23 (1.11-1.36)	NA	NA
Race × ADI, adjusted for age			
ADI <25	1 [Reference]	2.37 (1.88-2.98)	1 [Reference]
ADI 25-49	1.29 (1.15-1.46)	1.47 (1.29-1.69)	1 [Reference]
ADI 50-74	1.73 (1.52-1.96)	1.21 (1.07-1.36)	1 [Reference]
ADI ≥75	1.95 (1.67-2.73)	1.15 (0.99-1.32)	1 [Reference]
Black race, main effect size	2.19 (1.77-2.73)	NA	NA
ADI <25 × Black	1 [Reference]	NA	NA
ADI 25-49 × Black	0.66 (0.51-0.85)	NA	NA
ADI 50-74 × Black	0.56 (0.43-0.71)	NA	NA
ADI ≥75 × Black	0.53 (0.41-0.70)	NA	NA
Race × ADI, fully adjusted^b^			
ADI <25	1 [Reference]	1.92 (1.45-2.55)	1 [Reference]
ADI 25-49	1.21 (1.06-1.38)	1.11 (0.92-1.35)	1 [Reference]
ADI 50-74	1.29 (1.11-1.50)	1.19 (1.03-1.40)	1 [Reference]
ADI ≥75	1.33 (1.06-1.66)	1.11 (0.92-1.36)	1 [Reference]
Black race, main effect size	1.69 (1.31-2.29)	NA	NA
ADI <25 × Black	1 [Reference]	NA	NA
ADI 25-49 × Black	0.66 (0.49-0.93)	NA	NA
ADI 50-74 × Black	0.70 (0.52-0.95)	NA	NA
ADI ≥75 × Black	0.75 (0.54-1.06)	NA	NA

Abbreviations: ADI, area deprivation index; HR, hazard ratio; NA, not applicable.

^a^ The lowest ADI quartile (<25) indicates the lowest level of neighborhood deprivation; the highest ADI quartile (≥75) indicates the greatest level.

^b^ The fully adjusted model was a multivariable Cox proportional hazards regression model controlling for available clinicopathological factors and hospital differences, including age at diagnosis, cancer stage at diagnosis, tumor grade at diagnosis, hormone receptor type at diagnosis, treatment course, and hospital. When stratifying by Black or White patients, all variables except the race variable remained in the model; when stratified by ADI group, all variables except for ADI category remained in the model.

The interaction associations between ADI category and race were significant. As seen in Table 2, the HRs increased for White women as the ADI quartiles increased (HR for ADI of 25-49, 1.22 [95% CI, 1.07-1.39]; HR for ADI of 50-74, 1.32 [95% CI, 1.13-1.53]; and HR for ADI ≥75, 1.33 [95% CI, 1.07-1.65]), a pattern that also was observed in the models without the interaction. However, the same pattern was not observed among Black women, in which there was no apparent benefit of living in less deprived areas (quartile 25 to 49: HR, 0.81 [95% CI, 0.61-1.07]; quartile 50 to 74: HR, 0.91 [95% CI, 0.70-1.18]; and quartile ≥75: HR, 1.05 [95% CI, 0.70-1.36]). Among Black women, the overall association with mortality was significantly greater than among White women, as demonstrated by the main association of race combined with the HRs of race × ADI. The main effect size of being Black and living in the lowest ADI quartile was an HR of 1.69 (95% CI, 1.31-2.29). Among White women, the mortality hazard of living in an area with an ADI of 25 to 49 compared with less than 25 was 1.29 (95% CI, 1.11-1.50); for Black women, this hazard of moving into the group with an ADI of 25 to 49 was reduced at a rate of 0.66 (95% CI, 0.49-0.93). The interaction effect of being Black and moving to the most deprived ADI group compared with being White and moving to the most deprived ADI group was not statistically significant in the fully adjusted interaction model, emphasizing that Black and White women were similar only in this most deprived area (HR, 1.11 [95% CI, 0.92-1.36]). The associations were clearer when comparing the HRs of Black women vs White women in data stratified by ADI group, as seen in the far-right columns of the interaction model rows in Table 2. For example, when stratifying into the lowest ADI group, Black women had an HR of 2.37 (95% CI, 1.88-2.98) compared with White women. Black and White women did not have statistically different risk at the highest ADI level, however (HR 1.15 [95% CI, 0.99-1.32]). The interpretation is complex, but the overall result suggests that (1) Black women with breast cancer had higher mortality than White women with breast cancer, and (2) ADI had a different association with mortality among Black and White women.

**Figure 1.**
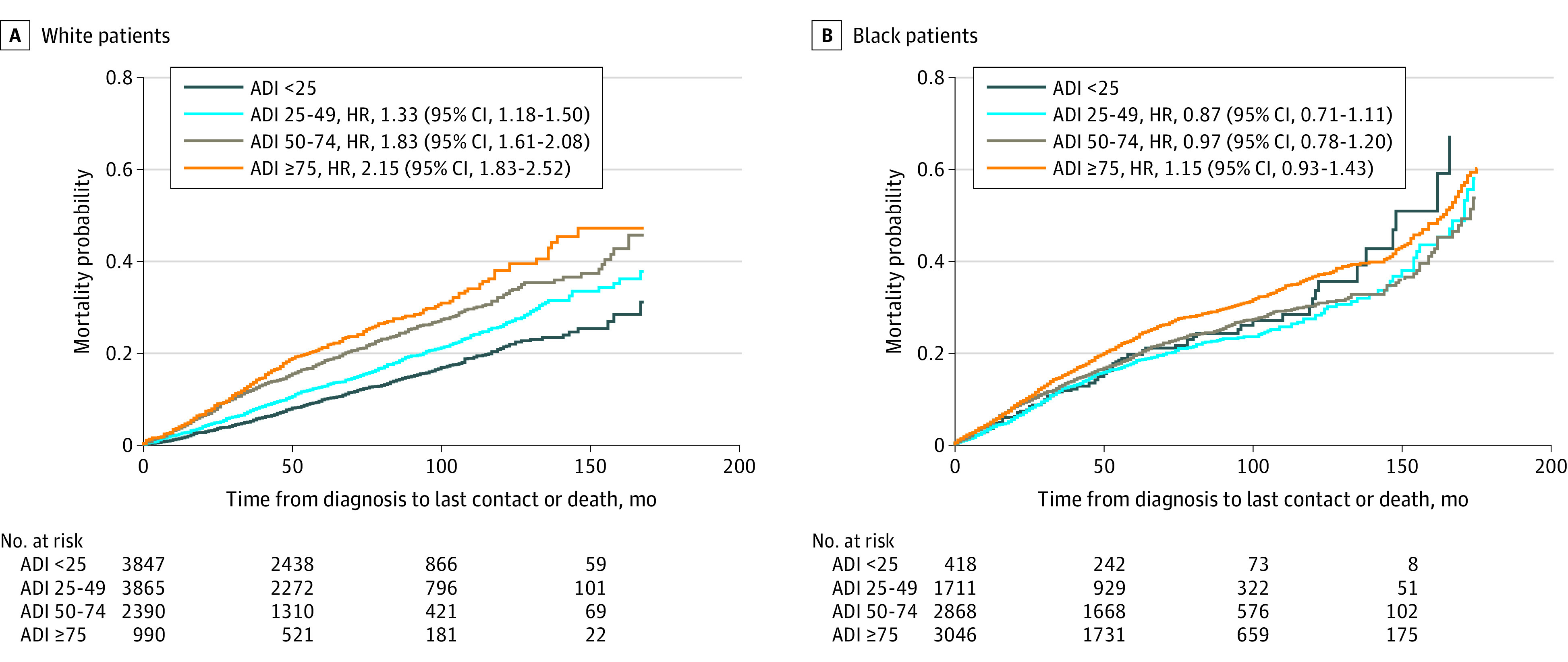
Associations Between Area Depriviation Index (ADI) and Probability of Patient Mortality for Black and White Patients With Breast Cancer Data are shown as Kaplan-Meier curves. The lowest ADI quartile (<25) indicates the lowest level of neighborhood deprivation; the highest ADI quartile (≥75), the greatest level. HR indicates hazard ratio.

**Figure 2.**
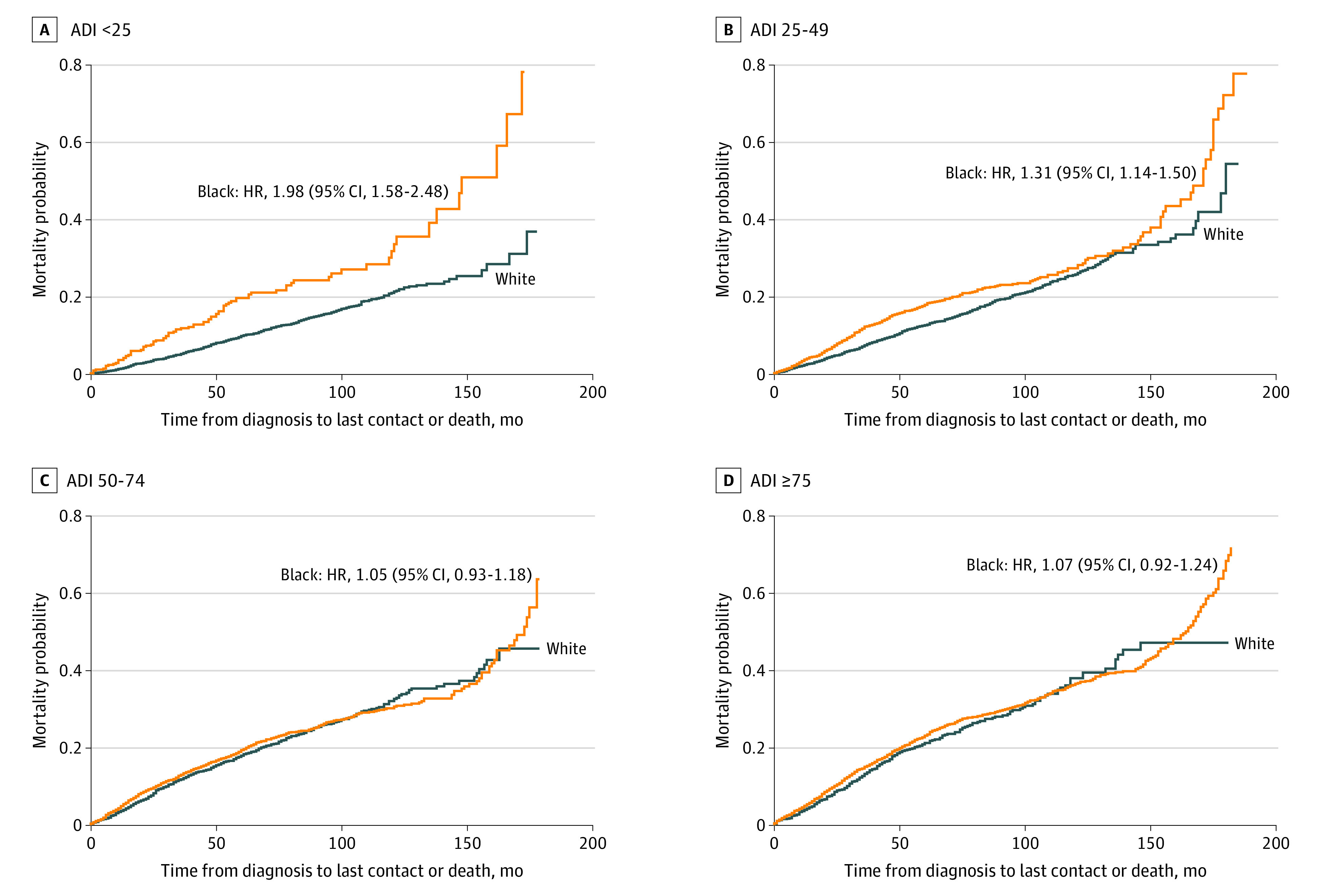
Association Between Race and Mortality Across All Area Deprivation Index (ADI) Categories Kaplan-Meier curves depict mortality probability for Black and White women stratified by ADI group. The lowest ADI quartile (<25) indicates the lowest level of neighborhood deprivation; the highest ADI quartile (≥75), the greatest level.

To elucidate the disparate effects of ADI, the survival model was estimated separately for each racial group. For White women, the hazard of increasing ADI on mortality was strong, sequential, and in the expected direction: groups in greater ADI quartiles had higher rates of mortality. For Black women, no ADI group conferred significant changes in the proportional hazard for mortality compared with those living in the least deprived group. These trends were visualized by Kaplan-Meier curves (Figure 1), with mortality curves plotted for each ADI group by race. Figure 1A presents the curves for White women, which show clear separation from each other and reveal higher mortality odds as one moves to higher ADI groups. Figure 1B shows the ADI group curves for Black women, which largely intersect each other over time, indicating that all ADI groups have similar survival rates. Figure 2 presents mortality curves for Black and White women stratified by the 4 ADI groups. Figure 2A and B highlight a significant difference in mortality between Black and White women in the low (ADI <25) and moderate (ADI, 25-49) deprivation groups: Black women had a significantly increased mortality rate compared with White women among those residing in less deprived neighborhoods (HRs, 1.98 [95% CI, 1.58-2.49] and 1.31 [95% CI, 1.14-1.50], respectively). However, in the groups with higher deprivation (ADI 50-74 and ≥75), there was no difference between Black and White women (HRs, 1.05 [95% CI, 0.93-1.18] and 1.07 [95% CI, 0.92-1.24], respectively) (Figure 2C and D).

**Figure 3.**
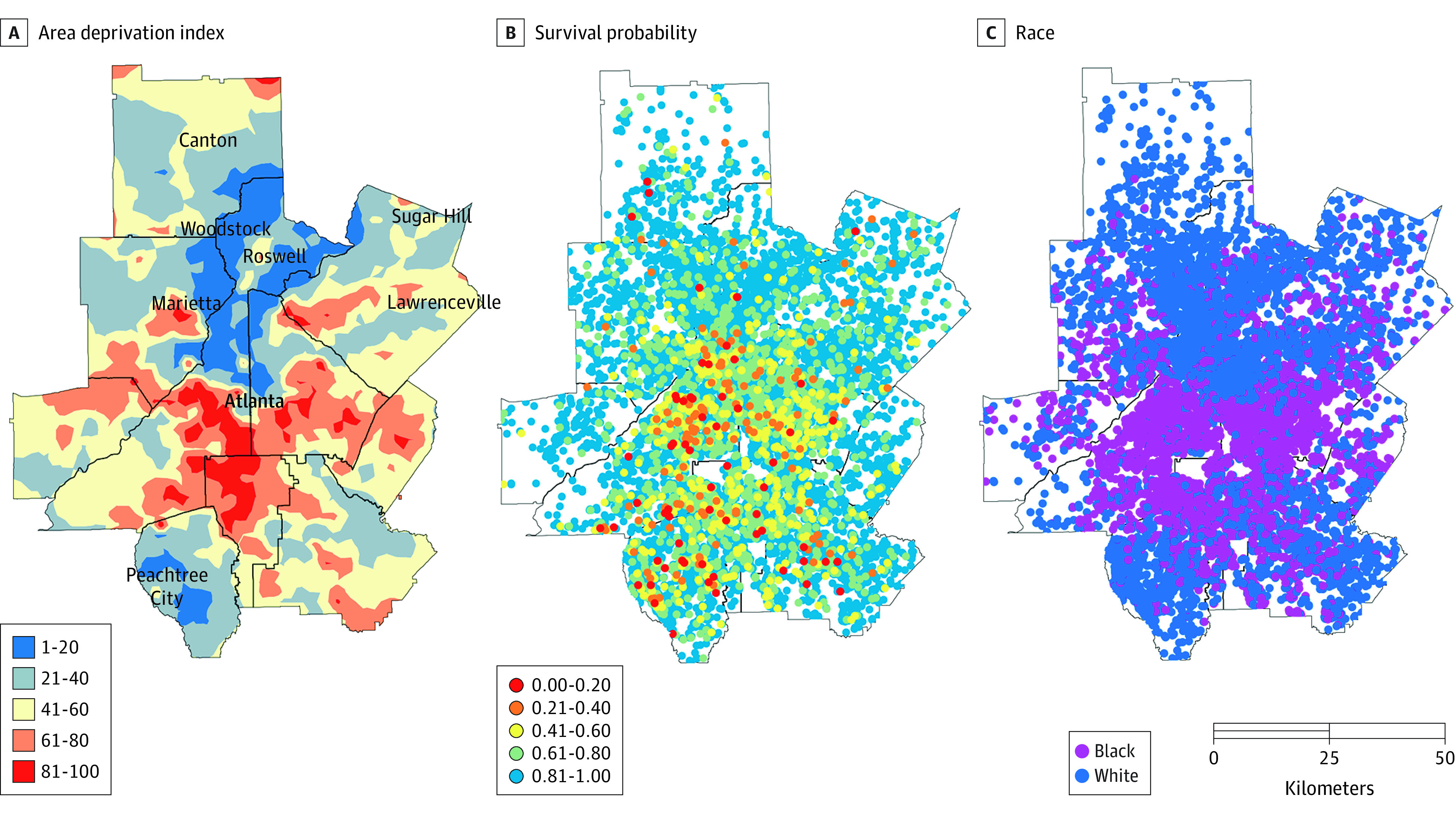
Visualization of Area Deprivation, Survival Probability, and Patient Race Mapping area deprivation, survival probability, and patient race revealed pronounced geographic disparities and spatial coincidence among these 3 variables. The lowest area deprivation index quartile (<25) indicates lowest level of neighborhood deprivation; the highest area deprivation index quartile (≥75), greatest level.

Mapping ADI, survival probability, and patient race revealed pronounced geographic disparities and spatial coincidence among these 3 variables. A comparison of the maps revealed that high ADI areas had greater Black patient density and low survival probability. In the 10-county metropolitan Atlanta region (Figure 3), where a large proportion (73.0%) of the patient cohort lived, the most deprived area (represented by high ADI values) was south Atlanta, and patients in this area had a low survival probability. For example, high ADI is represented by red, orange, and yellow hotspots in Figure 3A; these spots aligned with lower estimated survival probabilities, shown in Figure 3B as red, orange, or yellow points on the map. Figure 3C depicts locations of patients by race, with Black women (purple dashes) aligned with lower survival and higher ADI.

## Discussion

In this cohort study, we assessed associations of ADI and race with mortality among patients with breast cancer diagnosed in Georgia. We found that across the different ADI strata except for the most deprived areas, Black patients were more likely to die of breast cancer than White patients; in the most deprived neighborhoods, mortality rates were approximately the same. Importantly, we found that race modified the association of ADI with mortality in unexpected ways. As expected, White patients living in low ADI areas showed considerably lower rates of breast cancer mortality compared with White patients living in deprived neighborhoods. However, the same did not hold true for Black patients, who showed similar rates of mortality across all levels of deprivation. In other words, in multivariable-adjusted models, ADI was associated with breast cancer mortality among White patients but not among Black patients.

This unexpected pattern of findings suggests that additional factors related to neighborhoods may not be fully captured by the ADI as an aggregate indicator of neighborhood deprivation. Built environment factors (eg, green spaces and recreational facilities), food landscape (eg, proximity to grocery stores, healthy foods), unhealthy amenities (eg, fast food restaurants, smoke shops, alcohol outlets), and access to health care (eg, proximity to hospitals) are not captured by the ADI. Numerous other metrics of area-level factors are associated with broad health disparities, such as residential segregation and lending bias, which contribute to systematic disparities in housing quality.^41^ Evidence suggests that these factors are associated with increased breast cancer mortality.^42^

Our findings—that Black patients living in less-deprived neighborhoods have higher breast cancer mortality than similarly situated White patients—align with the diminishing returns hypothesis, which posits that members of racial minority groups do not attain the same health returns from high SES (associated with low ADI) as their counterparts from nonminority groups.^43,44,45,46^ For example, for breast, colorectal, and prostate cancers, Kish et al^46^ found that survival disparities between non-Hispanic Black and non-Hispanic White patients not only persisted across SES strata but grew larger among higher-SES groups (adjusting for stage, age, and treatment course). One possible explanation for these disparities could be persistent discrimination (social and economic) against Black individuals even as they improve their individual SES. Indeed, prior research has demonstrated that Black individuals whose income moderately increased over time were more likely to experience acute and chronic discrimination than those who remained within the same SES group.^43,44,45,46,47^

Another possible source of racial disparities in breast cancer mortality may be limited and delayed access to cancer screening and treatment in low-SES neighborhoods, because early detection is an important determinant of breast cancer survival.^48,49^ Although breast cancer risk is similar between Black and White women,^50^ Black women are more often diagnosed at advanced stages of disease, which contributes to their higher mortality rates.^51^ Results from our data set validate this finding. A recent population-based cohort study of patients with triple-negative breast cancer found that the risk of breast cancer mortality, which was significantly higher in Black women compared with White women, was partially explained by disparities in receipt of surgery and chemotherapy.^52^ In our sample, we observed a younger age at presentation in Black patients across breast cancer subtypes. This factor may be ameliorated by adapting current screening guidelines to recommend that Black women be screened at a younger age. Similarly, there are treatment disparities across the board for Black women, who receive cancer treatments inferior to those received by White women with similar cancer presentations.^48^ Both late diagnosis and disparities in treatment may be related to implicit bias among clinicians, which has been shown to lead to less adequate cancer treatment for members of minority groups in the US.^53^

As we expected, both Black and White patients with breast cancer living in the most deprived areas (which lack resources and have poor health care access) experienced similarly poor prognoses in terms of mortality. However, we expected that racial disparities in mortality would be explained by the fact that more Black women lived in deprived areas and that these disparities would not be pronounced after controlling for ADI. This outcome was not the case, because less deprivation was not associated with lower mortality for Black women. Based on our findings and previous evidence, we hypothesize that these disparities in outcomes may, in part, be explained by chronic stress and allostatic load. Chronic stress can trigger epigenetic modifications that persist even when the stressor is ameliorated, even across generations.^11,54,55,56,57,58,59,60,61,62,63,64,65,66,67,68,69,70,71^ Moving to a less deprived neighborhood later in life would thus not immediately reverse the consequences of prior experiences with fewer resources. However, our data do not contain historic living conditions for patients, but only their last known address; we therefore can make no claims about this, for some Black women in our study cohort may have lived in less-deprived areas all their lives. This is a promising line of inquiry: further research is needed on the possible epigenetic changes brought about by past and present neighborhood deprivation and which of these changes may lead to worse breast cancer prognoses.^72^ Understanding the interplay among race, SES, area-level deprivation during the life course, and breast cancer outcomes may lead to better intervention strategies for narrowing the disparity gap.

### Limitations

This study has some limitations. First, the study design precludes causal conclusions regarding the risk of disease. Second, we were unable to directly investigate Hispanic ethnicity as a subgroup owing to limitations in data availability. Third, varying degrees of data were missing on some of the controlling covariates, and some cases were therefore excluded from the survival analyses. The patients removed from the fully adjusted model owing to missing data tended to be Black, in a high ADI group, and dead, so the potential for reduced statistical power and small biases in estimates exists owing to fewer outcome events. However, the direction of association and inferential conclusions were consistent across the fully adjusted model and the unadjusted or partially adjusted models (which did not remove as many missing cases), providing confidence in the overall conclusions.

In addition, breast cancer is a complex disease that is influenced by multiple factors, including social and economic factors. We did not have complete information on patients’ economic and social histories and current experiences. In future studies, detailed patient histories regarding housing location, employment, income, health insurance, and perceived discrimination would better elucidate nuances in social and economic factors associated with breast cancer outcomes.

We did not have information about individual-level SES, which may mitigate the association of ADI with breast cancer mortality. Furthermore, ADI is not a complete measure of deprivation. Area-level SES measures such as ADI may insufficiently capture deprivation and other factors in health disparities across different racial subpopulations^73,74^; they do not measure additional neighborhood characteristics that profoundly influence health outcomes, such as walkability, availability of healthy food, health care access, racial residential segregation, and crime rate.^75^ Still, these findings can contribute to our understanding of how area deprivation contributes to health outcomes and help shape community-level interventions and policy changes.

## Conclusions

The findings of this cohort study suggest that there are significant racial differences in the association between ADI and breast cancer mortality. Our data suggest that Black women are more likely to live in high-deprivation areas, and higher deprivation is associated with unfavorable breast cancer outcomes. These data explain some of the differences in breast cancer outcomes between Black and White women. However, ADI alone was insufficient to explain the observed racial disparities. Among Black women with breast cancer, ADI had no association with overall mortality; Black women from all ADI strata had similar outcomes. In contrast, White patients with breast cancer had higher survival rates as deprivation decreased. Future research should seek to better understand other factors contributing to racial disparities in breast cancer mortality, especially among patients living in the least deprived areas, as well as the possible link between breast cancer mortality, socioeconomic deprivation, and epigenetic influence. Additional study may help identify modifiable factors and inform intervention strategies to attenuate these racial disparities in breast cancer outcomes.

## Notes

Supplement. **eMethods.** Missing Data
eReference
