# Supplementary material for: Association of Race and Area Deprivation With Breast Cancer Survival Among Black and White Women in the State of Georgia
Source: JAMA Netw Open. 2022 Oct 28;5(10):e2238183. doi: 10.1001/jamanetworkopen.2022.38183 (PMC9617173; doi:10.1001/jamanetworkopen.2022.38183)
Supplement: Supplement. — eMethods. Missing Data eReference [file jamanetwopen-e2238183-s001.pdf]

Supplementary Online Content

Luningham JM, Seth G, Saini G, et al. Association of race and area deprivation with breast cancer survival among Black and White women in the state of Georgia. *JAMA Netw Open*. 2022;5(10):e2238183. doi:10.1001/jamanetworkopen.2022.38183

eMethods. Missing Data

eReference

This supplementary material has been provided by the authors to give readers additional information about their work.

© 2022 Luningham JM et al. *JAMA Network Open*.

eMethods. Missing Data

Patient records were included based on availability of home address, race, vital status, and dates of diagnosis and last contact. However, some patient records contained missing data on clinical/pathological variables which were included as covariates in the fully adjusted survival model. In total, 3,320 patients had missing information on at least one variable included in the model. There were differences in missingness by patient race, ADI, and health care system. This missingness was primarily driven by high rates of missing data in the Grady Health System. Grady Hospital is a public hospital that is supported by the local counties and serves many low-income patients (likely to reside in higher ADI areas). In our sample, Grady patients were disproportionately Black and 80% resided in the two highest ADI groups. Additionally, 72% of the Grady patient records contained missing data, most commonly missing hormone subtype, stage, treatment course, or tumor grade. Within Grady patients alone, Race was not correlated with missingness—90% of patients with missing data were Black, and 87% of patients with complete records were Black. Piedmont Healthcare System provided a small proportion (9%) of patient records with missing data. The primary source of missing data was missingness on breast cancer stage (86%). Within the Piedmont system, 44% of patients with missing data were Black, whereas 31% of patients with complete records were Black. Emory supplied only complete records for all required variables. There was a small association between vital status and missing data. For patients with complete records, about 16% were deceased. Conversely, 35% of patients with some missing data in their records were deceased.

The data do not meet the assumptions of the Missing Completely at Random mechanism (Rubin, 1976) because the missingness process is associated with values of particular variables (e.g., hospital = Grady, vital status = deceased). However, we assumed that the Missing at Random mechanism was satisfied, which specifies that the conditional

© 2022 Luningham JM et al. *JAMA Network Open*.

distribution of the missingness process does not depend on the missing values in the data after conditioning on the observed values in the data.

© 2022 Luningham JM et al. *JAMA Network Open*.

eReference.

Rubin DB. Inference and missing data. Biometrika. 1976;63(3):581-592.

© 2022 Luningham JM et al. *JAMA Network Open*.
